# Immune checkpoint inhibition alters patterns of failure in inoperable stage III non-small cell lung cancer patients treated with chemoradiotherapy

**DOI:** 10.1007/s00432-025-06355-y

**Published:** 2025-11-01

**Authors:** Julian Taugner, Silja Stamer, Kerstin Hofstetter, Chukwuka Eze, Lukas Käsmann, Kerstin Clasen, Philipp Hartig, Werner Spengler, Thorben Groß, Farkhad Manapov, Claus Belka, Maximilian Niyazi

**Affiliations:** 1 Department of Radiation Oncology, University Hospital Tuebingen, Tuebingen, Germany; 2 Department of Radiation Oncology, University Hospital, LMU Munich, Munich, Germany; 3 Department of Medical Oncology and Pneumology (Internal Medicine VIII), University Hospital Tuebingen, Tuebingen, Germany; 4 National Center for Tumor Diseases (NCT), Partner Site Tuebingen, Tuebingen, Germany; 5 German Cancer Consortium (DKTK), Partner Site Munich, Munich, Germany; 6 Radio-LOG MVZ Guenzburg, Guenzburg, Germany

**Keywords:** Lung cancer, NSCLC, Chemoradiotherapy, Immunotherapy, Patterns-of-failure

## Abstract

**Purpose:**

We compared failure patterns in patients with inoperable stage III non-small cell lung cancer (NSCLC) treated with chemoradiotherapy (CRT) alone versus CRT combined with sequential and/or concurrent immune checkpoint inhibitors (CRT-IO).

**Methods:**

Retrospective real-world data from 221 patients across two German tertiary cancer centers were analyzed. Of these, 74 received CRT-IO, including sequential durvalumab (85%) and concurrent/sequential nivolumab (15%), while 148 received CRT alone. First failure site and time to failure were compared.

**Results:**

Between 2012 and 2022, all patients received thoracic radiotherapy (≥ 60 Gy) and at least two cycles of platinum-based chemotherapy. Induction chemotherapy was administered in 36%, and induction chemo-immunotherapy in 2%. Median follow-up was 51.7 months (95% CI 47.0–56.4). Median overall survival (OS) for the entire cohort was 37.1 months (95% CI 26.0–48.2), with OS in the CRT-IO group not reached vs. 27.1 months (95% CI 18.5–25.7) in the CRT group (*p* < 0.001). Median progression-free survival (PFS) was 22.8 months (95% CI 6.4–39.1) for CRT-IO versus. 9.9 months (95% CI 7.0–12.8) for CRT (*p* = 0.001, see Fig. 1).

Failure patterns differed significantly. CRT-IO patients had lower loco-regional progression (LRP) rates (9.5% vs. 21.8%, *p* = 0.023) and were more frequently alive without progression (45.9% vs. 16.3%, *p* < 0.001). Brain metastasis (BM) as the first failure, multifocal progression (MFP) and isolated extracranial distant metastasis (ecDM) rates were comparable between the CRT and CRT-IO subgroup. Women had a higher risk of isolated BM (17.3% vs. 6.8%, *p* = 0.016), whereas squamous cell carcinoma (SCC) patients had higher LRP rates (25.3% vs. 13.0%, *p* = 0.016). Median post-progression survival (PPS) was 19.4 months (95% CI 16.8–22.0) for CRT-IO and 9.5 months (95% CI 5.8–13.1) for CRT (*p* = 0.207). PPS was longer after BM (19.9 months) vs. LRP (8.5 months, *p* = 0.076) and significantly better in women (20.7 vs. 8.9 months, *p* = 0.012) and adenocarcinoma/non-otherwise-specified-carcinoma (AC/NOS) vs. SCC (*p* < 0.001).

**Conclusion:**

CRT-IO significantly improves OS, PFS, and LRP control compared to CRT alone. Failure patterns and survival disparities by histology and gender suggest tailored surveillance and treatment strategies are needed. Further studies should optimize management of LRP and long-term outcomes in CRT-IO-treated patients.

**Supplementary Information:**

The online version contains supplementary material available at 10.1007/s00432-025-06355-y.

## Introduction

In patients with inoperable stage III non-small cell lung cancer (NSCLC), the vast majority experience loco-regional and distant recurrences within the first two years following completion of primary treatment (Auperin et al. 2010; Flentje et al. 2016; O'Rourke et al. 2010; Sause et al. 1995; Vokes et al. 2007). Chemoradiotherapy (CRT) has been the standard of care for over three decades (Auperin et al. 2010). Since 2017, maintenance therapy with the programmed cell death ligand 1 (PD-L1) inhibitor durvalumab has been recommended following CRT after the pivotal PACIFIC-trial (Antonia et al. 2018, 2017), starting the era of CRT combined with maintenance immune checkpoint inhibition (CRT-IO). However, despite these advancements, the majority of patients will still face progressive disease experiencing multifocal progression (MFP), isolated brain metastasis (BM), isolated extracranial distant metastasis (ecDM) or isolated loco-regional progression (LRP).

An intensified follow-up strategy, incorporating computed tomography (CT) and 18F-fluorodeoxyglucose positron-emission tomography/CT (FDG-PET/CT), may enable the earlier detection of asymptomatic disease progression post-CRT and potentially enhance post-recurrence survival outcomes and is therefore recommended (Cerfolio et al. 2004; Lardinois et al. 2003; Schneider et al. 2020; Westeel et al. 2022). Disease progression following primary treatment is strongly associated with a substantial decline in both quality of life and overall survival [12–16]. Furthermore, previous studies have demonstrated that the time to loco-regional recurrence and the onset and distribution of distant metastases (DMs) significantly influence patient prognosis [17].

We have previously reported on failure patterns in patients treated with CRT alone and compared the onset of LRP and metastasis between those receiving CRT versus CRT-IO (Florsch et al. 2023; Hofstetter et al. 2024; Taugner et al. 2020; Unterrainer et al. 2022). In this study, we aim to analyze data from two high-volume tertiary cancer centers in Germany to assess shifts in failure patterns following the introduction of CRT-IO. Additionally, we plan to present data on salvage treatment options, including post-progression survival outcomes, for the patients of this cohort in the near future.

## Patients and Methods

We screened 398 consecutive patients with unresectable stage IIIa-IIIc NSCLC (UICC 8th edition) treated between 2011 and 2023. Of these, 221 (55.5%) were treated with two cycles of concomitant platinum-based chemotherapy and 60–70 Gy of thoracic radiotherapy (TRT) and therefore included in the analysis. Baseline FDG-PET/CT was performed in 218 (98.6%) patients prior to the initiation of multimodal treatment to enhance contouring accuracy. Cranial contrast-enhanced magnet resonance imaging (cMRI) was conducted in 105 (47.5%) patients, while the remaining patients underwent cranial contrast-enhanced CT. Next generation sequencing (NGS) or other forms of tumour mutation analysis were only performed in < 10% of patients and analysis concerning tumour driver mutations and failure-patterns, therefore could not be carried out. It is of note, that in both institutions all patients are required to undergo NGS prior to treatment since 2023. CRT was the recommended therapy of a multidisciplinary tumor-board for each patient. CRT-IO treatment regimens (n = 74) included either concurrent and maintenance therapy with the PD-1 inhibitor nivolumab as part of the phase II ETOP 6–14 NICOLAS study (n = 11 patients, 15%) or maintenance therapy with the PD-L1 inhibitor durvalumab, following the PACIFIC trial protocol (n = 63; 85%). TRT was planned using conventional planning CT and/or PET-CT. Patients were positioned supine with their arms placed overhead. Target volumes were defined based on internal protocols, closely aligning with the later-published guidelines of the European Society for Radiotherapy and Oncology-Advisory Committee on Radiation Oncology Practice (ESTRO-ACROP) (Nestle et al. 2018). All patients were treated with photon beams delivered by a linear accelerator, using cone-beam CT–based image guidance. Follow-up imaging CT scans were obtained every three months for the first two years after treatment, every six months for the next three years, and annually thereafter. PET/CT, cMRI and histological confirmation of progressive disease were not mandatory but carried out when deemed necessary.

Four possible outcomes were documented for each individual patient: death without documented progression, MFP, isolated BM first and isolated ecDM first. Patients who were alive, without progression in the last documented follow-up, were censored. Survival parameters were calculated from the last day of TRT, considering only the first site of failure we calculated progression free survival (PFS), time to MFP, time to BM, time to ecDM and time to LRP. Patient and treatment-related characteristics are shown in Table 1.

Data was analysed with the Kaplan–Meier method using the log-rank test and multivariable Cox-regression using SPSS version 28 (IBM; Armonk, New York, USA). This retrospective study and individual patient data analysis were approved by the two local ethics committees (Ref. No. 17–230 and 423/2024BO2).

## Results

All patients received at least two cycles of platinum-based doublet chemotherapy combined with thoracic radiotherapy (TRT) of 60–70 Gy. Most patients (66.5%) were male with a median age of 66.6 years at diagnosis. Disease stages IIIA, IIIB, and IIIC (UICC 8th edition) were present in 32.6%, 41.2%, and 26.2% of cases, respectively. Histologically, 48.9% had AC, 44.8% had SCC, and 6.3% had large-cell or NOS carcinoma.

A total of 147 patients (66.5%) received CRT alone, while 74 (33.5%) underwent CRT-IO. No significant differences were observed between the CRT and CRT-IO groups regarding gender, histology, or disease stage (see Table 1). Treatment was delivered with volumetric arc therapy (VMAT) in 164 (74.2%) of patients, with step and shoot IMRT in 22 (10%) of patients and wit 3D-conformal radiotherapy (3D-CRT) in 35 (15.8%) of patients.

**Fig. 1:**
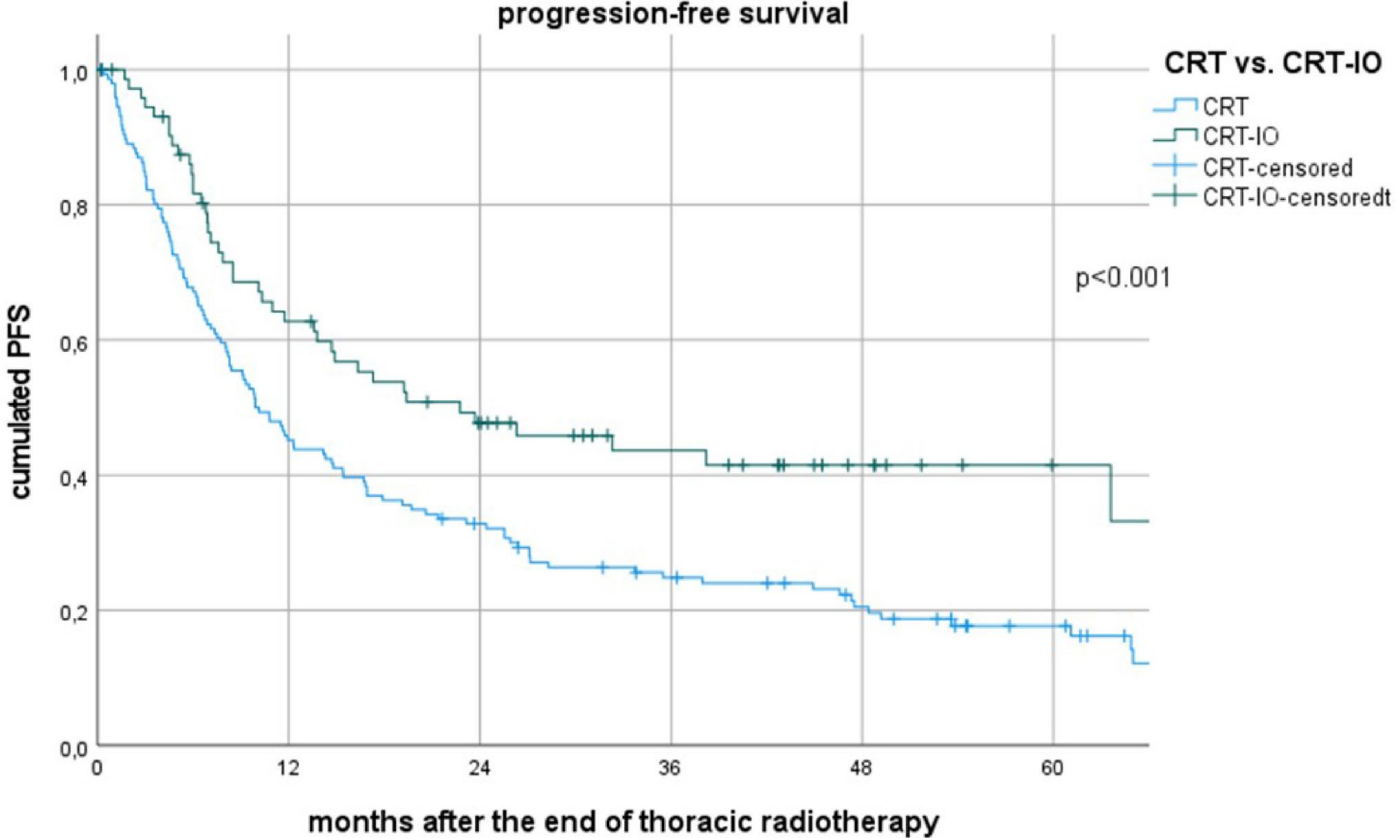
Progression free survival after the end of radiotherapy for patients treated with CRT vs CRT-IO

The median follow-up duration was 51.7 months (95% CI 47.0–56.4). Median OS and PFS for the entire cohort were 37.1 months (95% CI 26.0–48.2) and 12.3 months (95% CI 8.3–16.3), respectively. OS and PFS were significantly improved in the CRT-IO group compared to the CRT group. To date the OS has not been reached yet in the CRT-IO group vs. 27.1 months (95% CI 18.5–25.7) in the CRT group (*p* < 0.001), while median PFS was 22.8 months (95% CI 6.4–39.1) vs. 9.9 months (95% CI 7.0–12.8) (*p* = 0.001), see also Fig. 1.

Patients treated with VMAT hat significantly better OS (VMAT: 40.1, IMRT: 19.3, 3D-CRT: 19.1 months [*p* = 0.039]) and PFS (VMAT:14.8, IMRT: 7.4, 3D-CRT: 8.3 months [*p* = 0.003]) in the CRT alone-subgroup. All patients in the CRT-IO subgroup were treated with VMAT.

### Patterns of first failure

In the entire cohort, 13.1% of patients died without documented tumor progression. MFP occurred in 16.3%, BM as the first site in 10.4%, ecDM as the first site in 16.3%, and LRP in 17.6%. At the end of follow-up, 26.2% of patients remained alive without progression.

Progression patterns differed significantly between the CRT-IO and CRT subgroup: Patients receiving CRT-IO had a lower rate of death without documented progression (5.4% vs. 17.0%, *p* = 0.016) and were more likely to be alive without progression at the end of follow-up (45.9% vs. 16.3%, *p* < 0.001). CRT-IO also significantly reduced the incidence of LRP (9.5% vs. 21.8%, *p* = 0.023). However, rates of MFP, isolated BM, and ecDM did not differ significantly between groups. See Table 1.

Patients with UICC stage IIIA were significantly more likely to die without documented progression (*p* = 0.045) and showed a trend toward a lower incidence of BM as the first site of progression (*p* = 0.066). The likelihood of MFP (*p* = 0.555), ecDM (*p* = 0.982) and LRP (*p* = 0.444) did not correlate with UICC stage.

Women had a significantly higher risk of developing isolated BM first (17.3% vs. 6.8%, *p* = 0.016). Neither T-stage nor N-stage or delivery technique correlated significantly with failure patterns. Patients with SCC were significantly less likely to develop isolated BM first (2.0% vs. 21.0%, *p* < 0.001) but had a higher incidence of LRP as the first site of failure (25.3% vs. 13.0%, *p* = 0.016).

### Timing of first failure

**Fig. 2:**
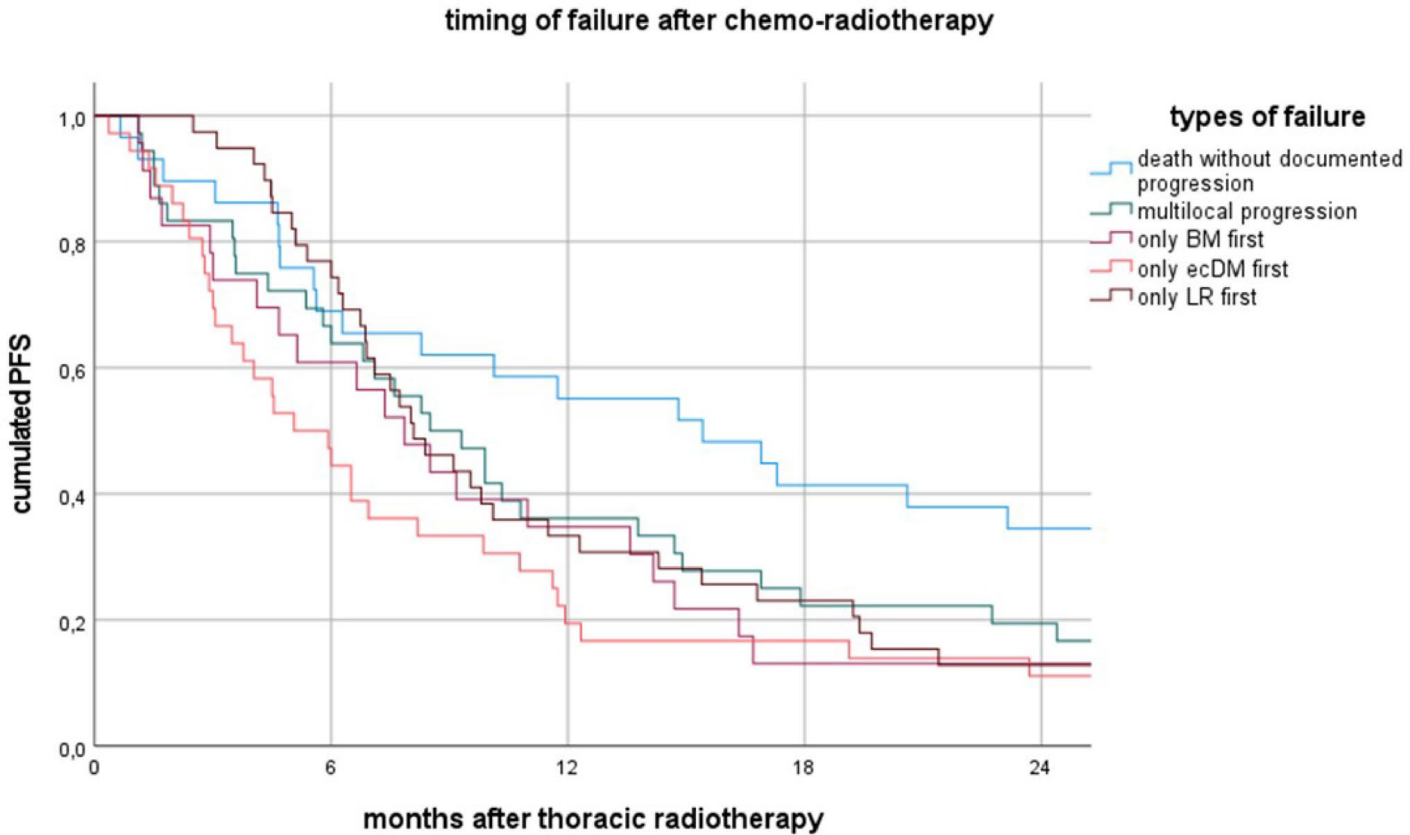
Timing of first failure after radiotherapy

The median time from the end of thoracic radiotherapy (TRT) to ecDM was 5.1 months (95% CI 2.9–7.3). BM occurred after a median of 7.9 months (95% CI 4.9–10.8), LRP after 8.1 months (95% CI 6.1–10.0), MFP after 8.5 months (95% CI 5.8–11.2), and death without documented progression after 15.4 months (95% CI 6.3–24.5). See also Fig. 2.

There was a trend toward a longer median time to BM in patients treated with CRT-IO compared to CRT alone (11.0 vs. 5.1 months, *p* = 0.082). Patients with large-cell or NOS carcinoma developed isolated BM significantly earlier (median 2.9 months) compared to those with SCC (6.6 months) and AC (8.5 months) (*p* = 0.016). Median time to LRP after TRT was significantly longer for AC (19.7 months, 95% CI 0.7–38.7) compared to SCC (8.0 months, 95% CI 6.4–9.6) and large-cell or NOS carcinoma (6.2 months, 95% CI 3.1–9.2) (*p* = 0.001).

Death without documented progression occurred significantly later in SCC (median 15.4 months, 95% CI 11.3–19.5) compared to AC (8.2 months, 95% CI 0.0–33.7) and was not observed in large-cell or NOS carcinoma (*p* = 0.028).

A trend toward a faster onset of LRP was observed in patients with UICC stage IIIC (*p* =* p* = 0.062) and cT4 tumours (*p* = 0.064). However, UICC stage, T-stage, N-stage, delivery techniques and gender had no significant effect on failure timing beyond these findings.

### Survival after first failure

Post-progression survival (PPS) was a median of 12.6 months (95% CI 5.5–19.7) overall, with 19.4 months (95% CI 16.8–22.0) in the CRT-IO group and 9.5 months (95% CI 5.8–13.1) in the CRT group (*p* = 0.207).

PPS varied by initial progression pattern: 18.6 months (95% CI 3.2–34.0) after MFP, 19.9 months (95% CI 10.5–29.5) after BM, 12.5 months (95% CI 0.0–26.0) after ecDM, and 8.5 months (95% CI 7.5–9.4) after LRP. Among patients initially treated with CRT-IO, those with BM as the first site of progression (n = 9), had not yet reached median PPS, whereas CRT-IO patients with LRP (n = 7) had a median PPS of only 2.4 months (95% CI 1.4–3.4, *p* = 0.076).

**Fig. 3:**
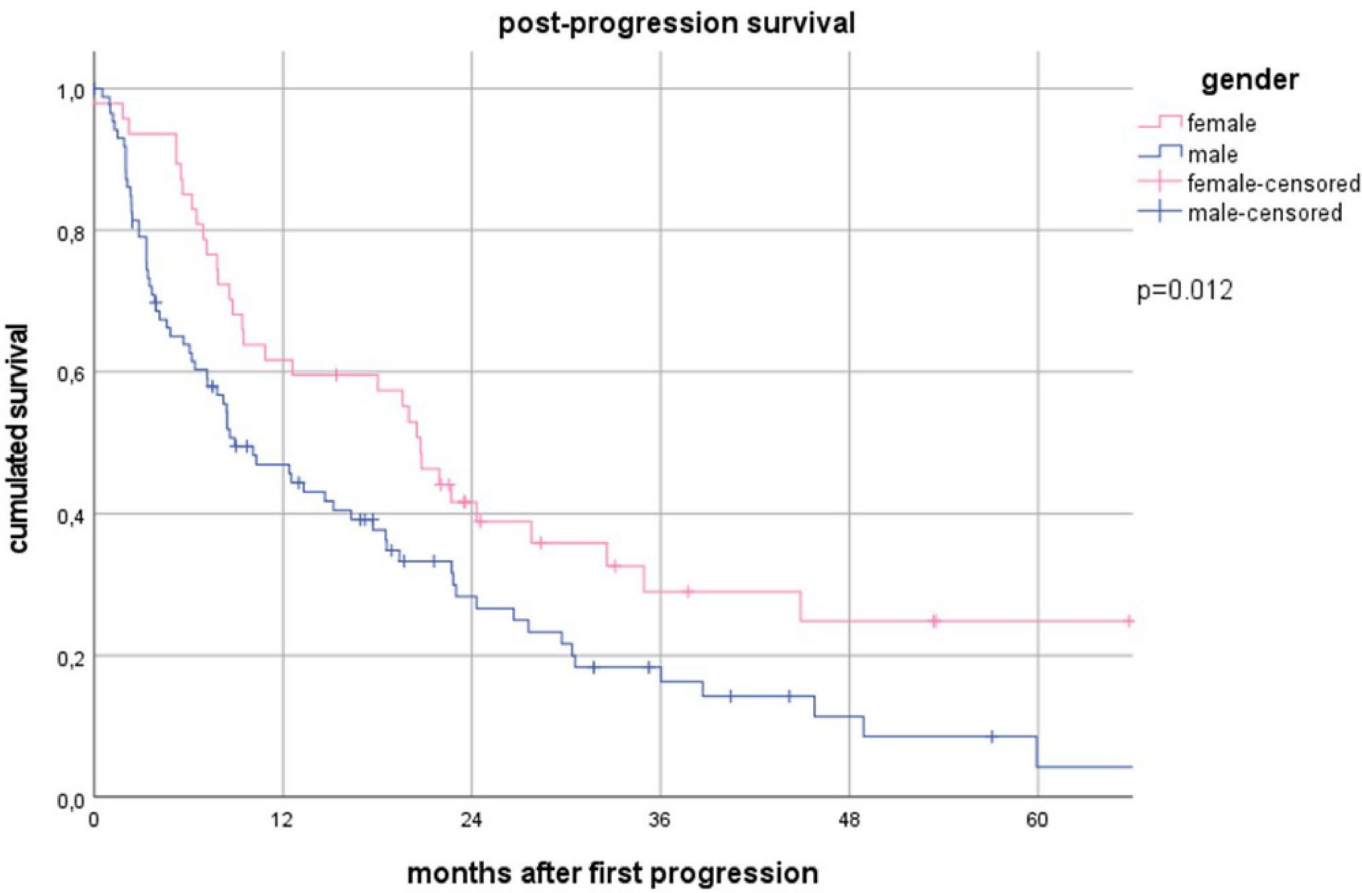
Post-progression survival after the first progression for female vs male patients

**Fig. 4:**
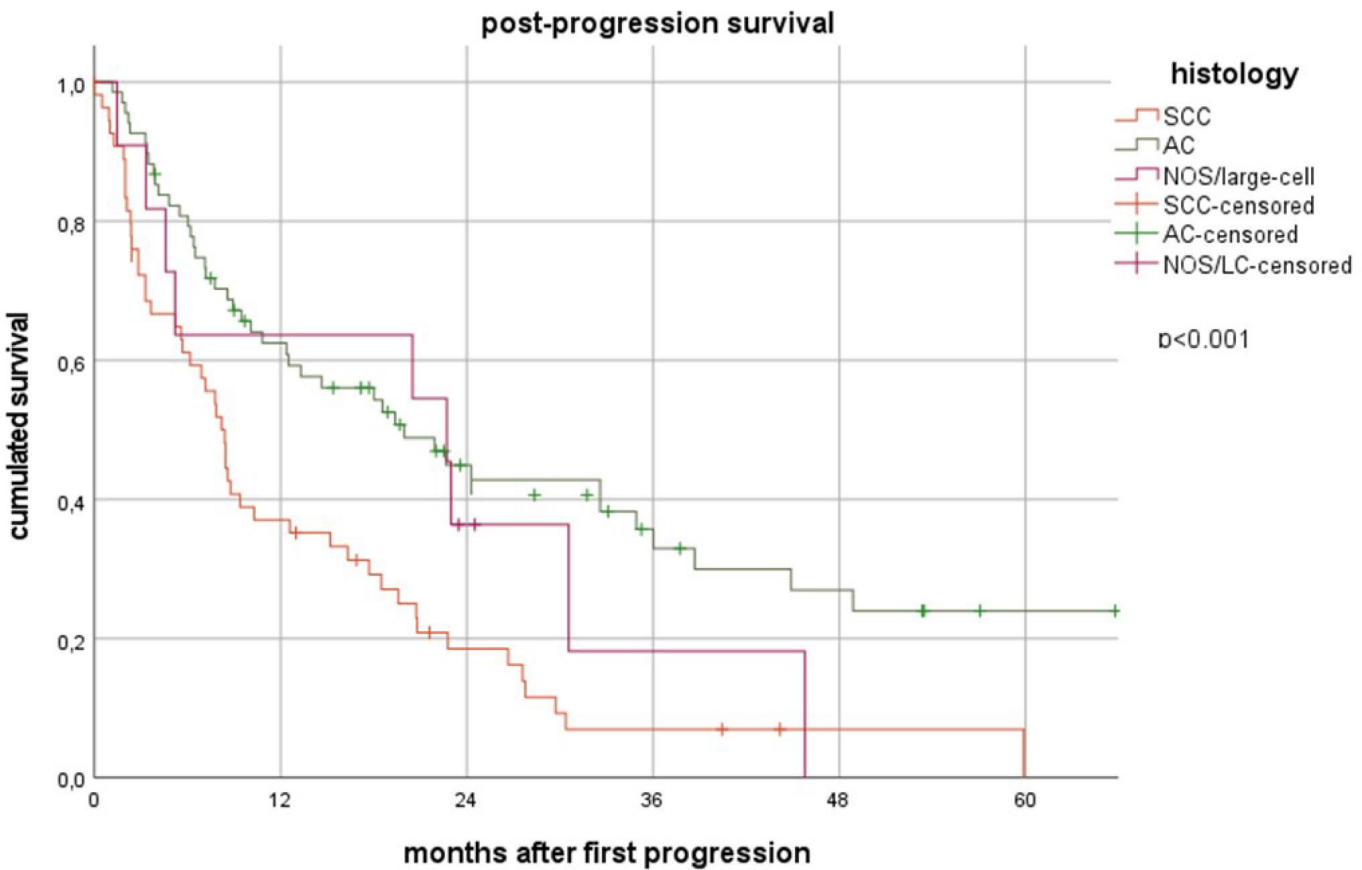
Post-progression survival after the first progression for patients with SSC vs Nos/large-cell vs AC

Women in general had significantly longer PPS than men (20.7 months [95% CI 17.7–23.8] vs. 8.9 months [95% CI 4.8–13.1], *p* = 0.012, Fig. 3). Patients with AC (20.0 months, 95% CI 11.0–28.9) and large-cell/NOS carcinoma (22.7 months, 95% CI 3.5–41.9) had significantly longer PPS than those with SCC (8.4 months, 95% CI 6.9–9.9, *p* < 0.001, Fig. 4).

Initial UICC stage and delivery technique had no significant impact on PPS.

### Gender and outcome

Female patients were more frequently diagnosed with AC (n = 45; 60%) and less frequently with SCC (n = 25; 33%) compared with male patients (AC: n = 63; 43%, SCC: n = 74; 51%). While PFS did not differ significantly between genders, OS was significantly longer in women than in men (46.7 months [95% CI 35.2–58.2] vs. 26.3 months [95% CI 16.1–36.5]; *p* = 0.045). Median PPS was also superior in women (20.7 months [95% CI 17.7–23.8] vs. 8.9 months [95% CI 5.0–13.1]; *p* = 0.012), and median time to death without documented tumor progression was significantly longer (27.7 months [95% CI 13.7–41.7] vs. 11.1 months [95% CI 6.9–15.3]; *p* = 0.011). A trend toward longer PPS following brain metastases was observed in women (*p* = 0.072). See also suppl. Table 2.

## Discussion

This study demonstrates that the addition of immunotherapy to concurrent chemoradiotherapy (CRT-IO) for patients with stage III NSCLC not only significantly improves survival, but also shifts patterns and timing of progression as well PPS survival. Compared to CRT alone, CRT-IO resulted in vastly prolonged median OS and PFS. The median OS was not reached in the CRT-IO cohort, whereas patients treated with CRT alone had a median OS of 27.1 months, reinforcing the efficacy of immunotherapy in this setting. Furthermore, CRT-IO significantly extended PFS (22.8 months vs. 9.9 months, *p* = 0.001), reflecting a reduced risk of disease progression especially in the first two years after CRT. These findings align with recent clinical trials, such as the PACIFIC trial, which established the benefit of consolidation immunotherapy following CRT in stage III NSCLC (Antonia et al. 2018, 2017; Flentje et al. 2016; Kakiuchi et al. 2024; Taugner et al. 2021).

The patterns of disease progression varied between treatment groups, with CRT-IO significantly lowering the rate of LRP compared to CRT alone (9.5% vs. 21.8%, *p* = 0.023). However, rates of MPF, BM, and ecDM were comparable between groups. The reduction in LRP may be attributable to the immunomodulatory effects of checkpoint inhibitors in irradiated areas, enhancing local tumor control after radiotherapy. Notably, a higher proportion of patients remained alive without progression in the CRT-IO group (45.9% vs. 16.3%, *p* < 0.001), underscoring the long-term benefits of adding immunotherapy to CRT. Furthermore, reduced toxicity due to improved delivery methods of thoracic-radiotherapy throughout the last decade might have considerably improved the overall outcome in both our patient subgroups compared with historic data (Auperin et al. 2010; Garg et al. 2014). This improvement is also reflected in our data with superior outcomes for patients treated with CRT alone when using the more advanced VMAT technique, as evidenced by significant improvements in overall survival (OS, *p* = 0.039) and progression-free survival (PFS, *p* = 0.003). Interestingly, no observable impact on failure patterns or PPS was noted based on whether patients were treated with VMAT, step-and-shoot IMRT, or 3D-CRT.

Reflecting the likelihood that never-smokers more often present with PD-L1–negative tumors, and considering that consolidation therapy with durvalumab is only permitted for PD-L1–positive tumors, it is unsurprising that the proportion of never-smokers is significantly higher in the CRT-alone subgroup compared to the CRT-IO subgroup (*p* = 0.032).

Subgroup analyses revealed additional prognostic factors influencing failure patterns. Women exhibited a significantly higher risk of BM as the first site of progression (17.3% vs. 6.8%, *p* = 0.016), a finding that aligns with prior research suggesting sex-based differences in NSCLC progression patterns (Kishi et al. 2023 ). Histological subtype also influenced failure patterns, with squamous cell carcinoma (SCC) being associated with a lower incidence of BM (2.0% vs. 21.0% in non-SCC, *p* < 0.001) but a higher rate of LRP (25.3% vs. 13.0%, *p* = 0.016). These findings suggest that histology-specific therapeutic strategies and aftercare procedures, such as tailored imaging, may be warranted to optimize treatment outcomes and detection of failure.

Zhu et al. developed a predictive model for first failure after definitive CRT in inoperable locally advanced NSCLC, incorporating smoking history, pathology, tumor location, EGFR mutation status, age, tumor diameter, clinical N stage, consolidation chemotherapy, and radiation dose (Zhu & Fu 2019; Zhu et al. 2020). Our findings largely align with this model, however, our cohort was more homogeneous, with all patients receiving similar radiation doses and chemotherapy. Due to limited mutation analysis, EGFR status remains unknown for most patients in our cohort. Because NGS is mandatory in both institutions since 2023 we hope to evaluate patterns of failure in patients with driver mutations in the near future. Unlike Zhu et al., we found no significant association between initial stage and failure patterns (Zhu & Fu 2019; Zhu et al. 2020). Numerous studies have reported that SCC patients have a higher risk of locoregional failure, whereas AC patients are more prone to distant failure (Katagiri et al. 2021; Kita et al. 2023; Nygard et al. 2018; Taugner et al. 2020). Oncogenic driver mutations must be considered in future personalized treatment as emphasized by the landmark LAURA study. (Lu et al. 2024). They must also be considered in personalized imaging approaches for follow-up care, as failure patterns differ significantly in these patients as reported by Nakamura et al. (Nakamura et al. 2019).

Regarding the timing of first failure, CRT-IO demonstrated a trend toward delaying BM occurrence compared to CRT alone (11.0 vs. 5.1 months, *p* = 0.082).This is consistent with findings reported by Yang et al. (Yang et al. 2023). The timing of LRP varied by histology, with adenocarcinoma (AC) exhibiting significantly longer median time to LRP (19.7 months) compared to SCC (8.0 months) and large-cell or NOS carcinoma (6.2 months) (*p* = 0.001). These findings indicate that histological subtype affects both the pattern and timing of failure, highlighting the importance of personalized follow-up and intervention strategies.

PPS analysis further highlights the survival advantage conferred by CRT-IO. With a median PPS of 12.6 months, PPS showed no significant differences between CRT-IO and CRT patients, even though CRT-IO patients had a numerically longer PPS than CRT-alone patients (19.4 vs. 9.5 months, *p* = 0.207). PPS varied significantly by failure site, with the longest survival observed following BM (19.9 months) and MFP (18.6 months), whereas LRP was associated with the poorest prognosis (8.5 months). Patients initially treated with CRT-IO who developed BM had not yet reached median PPS, whereas those with LRP had a particularly poor outcome (2.4 months, *p* = 0.076). These findings suggest that while systemic therapies may extend survival in patients with distant progression, those with LRP remain at high risk for poor outcomes. The reduced frequency of LRP in patients initially treated with CRT-IO may be a key factor why OS is improved this hugely. This is in line with our previously published data (Florsch et al. 2023; Hofstetter et al. 2024).

Interestingly, women had significantly longer PPS than men (20.7 vs. 8.9 months, *p* = 0.012), consistent with prior studies suggesting a survival advantage for female NSCLC patients (Taugner et al. 2019; Wakelee et al. 2006; Wheatley-Price et al. 2010). Histology also played a role in PPS, with patients with AC (20.0 months) and large-cell/NOS carcinoma (22.7 months) faring significantly better than those with SCC (8.4 months, *p* < 0.001). This is likely due to the higher incidence of LRP in SCC patients and the poor prognosis after onset of LRP. In contrast, the initial UICC stage did not significantly impact PPS, suggesting that the post-progression course is more strongly influenced by initial treatment and histological subtype than initial disease burden.

Female patients were more frequently diagnosed with ACC and less often with SCC than their male counterparts (*p* = 0.037), which may contribute to the higher incidence of brain metastases observed in this subgroup. Patients who developed BM demonstrated significantly longer PPS compared with those experiencing LRP, suggesting a potentially distinct disease pattern or the benefit of effective local therapies for intracranial disease. Moreover, among patients without documented progression, women had a significantly longer survival than men (27.7 vs. 11.1 months; *p* = 0.011). Taken together, these factors may help explain the significantly longer OS observed in women (46.7 vs. 26.3 months; *p* = 0.045), despite the absence of a significant difference in PFS. We additionally acknowledge that gender differences in outcomes may be influenced by immune-related phenomena, such as stronger innate and adaptive immune responses in females, as well as hormonal influences, particularly estrogen’s effects on immune function and the tumor microenvironment. D. P. Anobile et al. demonstrated that ERα expression transcriptionally upregulates CD274/PD-L1, with stronger effects observed in female tumors. Increased PD-L1 expression in ERα-high tumors was associated with local immune suppression and reduced infiltration of effective antitumor lymphocytes. These findings provide a rationale for combining aromatase inhibitors, such as letrozole, with pembrolizumab to potentially enhance therapeutic efficacy in NSCLC patients (Anobile et al. 2023). Y. Zhang et al. conducted a prospective observational cohort study measuring six plasma sex hormones prior to the first dose of immune checkpoint inhibitor (ICI) therapy in 61 patients with metastatic NSCLC. They correlated hormone levels with clinical benefit, progression-free survival (PFS), and overall survival (OS), finding that elevated pretreatment circulating levels of DHEA and 5-androstenediol (androgens) were associated with poorer ICI response outcomes (Anobile et al. 2023). Moreover, female patients are generally fitter and have a longer life expectancy than their male counterparts, which could contribute to the observed survival advantage. In the female cohort there was a numerically higher percentage of never smokers (13.3 vs 2.7%, *p* = 0.058). These factors warrant further investigation.

With increased salvage options, the PPS of patients with advanced NSCLC has steadily improved over the past decade, as demonstrated by the meta-analysis by Rutkowski et al. (Rutkowski et al. 2019). Our previous report in 2020 included only patients without durvalumab maintenance during initial treatment (Taugner et al. 2020). Given the established role of immunotherapy in first-line treatment, shifting failure patterns may now favor local ablative strategies for first failure after CRT-IO in inoperable locally advanced NSCLC (Friedes et al. 2024; Taugner et al. 2021; Xu et al. 2021).

This study has several limitations that should be acknowledged. Although the patient cohort was highly homogeneous with respect to baseline characteristics, tumor stage, and treatment indication, the data were collected over a period spanning more than a decade and across two high-volume institutions. Consequently, minor differences in treatment planning, radiation techniques, systemic therapy regimens, and follow-up procedures may have occurred, reflecting the evolution of clinical practice over time. A limitation of this retrospective analysis is the lack of detailed information on the rationale for induction therapy in all patients. In the German healthcare system, induction therapy is occasionally given at smaller hospitals before referral to tertiary centers for chemoradiotherapy to avoid delays. Some patients were initially considered for surgery but later declined or were deemed unresectable after induction chemotherapy, reflecting the complexity of real-world treatment pathways. Moreover, as with all retrospective analyses, our study is subject to inherent limitations, including potential selection bias, incomplete documentation, and unmeasured confounding variables that may have influenced treatment decisions and outcomes. The absence of randomization also restricts the ability to establish causal relationships between treatment strategies and survival endpoints.

Another important limitation is that the present analysis focused primarily on initial disease progression, and detailed data regarding salvage and palliative treatments following recurrence were not included. Given the complexity and heterogeneity of these management strategies, we believe that their comprehensive evaluation exceeds the scope of the current report. Nevertheless, we recognize that these factors play a crucial role in determining post-progression outcomes. To address this, we are currently curating and analyzing these data for a dedicated follow-up publication, which will provide an in-depth assessment of salvage and palliative treatment patterns and their impact on survival.

## Conclusion

In conclusion, this study supports the superior efficacy of CRT-IO over CRT alone in stage III NSCLC, particularly in improving OS and PFS while reducing LRP. Differences in failure patterns and PPS based on histology and gender highlight the need for tailored surveillance and treatment approaches. Further research should explore strategies to mitigate loco-regional recurrence and optimize long-term outcomes in patients receiving combined modality therapy.

## Supplementary Information

Below is the link to the electronic supplementary material.

Supplementary Material 1

Supplementary Material 2

## Data Availability

The datasets generated during and analysed during the current study are available from the corresponding author on reasonable request.
