# Supplementary material for: Immune checkpoint inhibition alters patterns of failure in inoperable stage III non-small cell lung cancer patients treated with chemoradiotherapy
Source: J Cancer Res Clin Oncol. 2025 Nov 1;151(12):313. doi: 10.1007/s00432-025-06355-y (PMC12579610; doi:10.1007/s00432-025-06355-y)
Supplement: Supplementary file 1 — Supplementary Material 1 [file 432_2025_6355_MOESM1_ESM.docx]

| **Patient and characteristics of first DM** | **Entire cohort**  **N (%)** | **female**  **N (%)** | **male**  **N (%)** | ***p* for CRT-IO vs. CRT** |
| --- | --- | --- | --- | --- |
| **Number of patients** (%) | 221 (100) | 75 (33.9) | 146 (66.1) |  |
| **CRT vs CRT-IO**  CRT (%) / CRT-IO (%) | 147 (66.5) / 74 (33.5) | 52 (69.3) / 23 (30.7) | 95 (65.1) / 51 (34.9) | *p=0.527* |
| **Histology**  SCC | 99 (44.8) | 25 (33.3) | 74 (50.7) | *p=0.037* |
| Adeocarcinoma | 108 (48.9) | 45 (60.0) | 63 (43.2) |  |
| Large cell/NOS | 14 (6.3) | 5 (6.7) | 9 (6.2) |  |
| **UICC-Stage**  IIIA | 72 (32.6) | 25 (33.3) | 47 (32.2) | *p=0.678* |
| IIIB | 91 (41.2) | 32 (42.7) | 59 (40.4) |  |
| IIIC | 58 (26.2) | 18 (24.0) | 40 (27.4) |  |
| **T-stage**  1  2  3  4 | 17 (7.7)  37 (16.7)  59 (26.7)  108 (48.9) | 3 (4.0)  17 (22.7)  23 (30.7)  32 (42.7) | 14 (9.6)  20 (13.7)  36 (24.7)  76 (52.1) | *p=0.603* |
| **N-stage**  0  1  2  3 | 28 (12.7)  16 (7.2)  93 (42.1)  84 (38.0) | 9 (12.0)  7 (9.3)  30 (40.0)  29 (38.7) | 19 (13.0)  9 (6.2)  63 (43.2)  55 (37.7) | *p=0.992* |
| **Pack-years**  median in years  never smokers | 40  14 (6.3) | 40  10 (13.3) | 40  4 (2.7) | *p=0.626*  *p=0.058* |
| **Treatment delivery**  3D-CRT  step-and-shoot IMRT  VMAT | 35 (15.8)  22 (10.0)  164 (74.2) | 18 (24.0)  5 (6.7)  52 (69.3) | 17 (11.6)  17 (11.6)  112 (67.7) | *p=0.136* |
|  |  |  |  |  |
| **Type of first failure** |  |  |  |  |
| Death without documented progression | 29 (13.1) | 9 (12.0) | 20 (13.7) | *p=0.725* |
| MFP | 36 (16.3) | 14 (18.7) | 22 (15.1) | *p=0.495* |
| BM | 23 (10.4) | 13 (17.3) | 10 (6.8) | *p=0.016* |
| ecDM | 36 (16.3) | 8 (10.7) | 28 (19.2) | *p=0.106* |
| LRP | 39 (17.6) | 12 (16.0) | 27 (18.5) | *p=0.647* |
| Censored (alive without progression) | 58 (26.2) | 19 (25.3) | 39 (26.7) | *p=0.826* |
|  |  |  |  |  |
| **Timing of Failure** | **months (95%CI)** | **months (95%CI)** | **months (95%CI)** |  |
| median follow-up | 51.7 (47.0-56.4) | 58.3 (46.0-70.5) | 48.9 (43.3-54.4) | *p=0.175* |
| median OS | 37.1 (26.0-48.2) | 46.7 (35.2-58.2) | 26.3 (16.1-36.5) | *p=0.045* |
| median PFS | 12.3 (8.3-16.3) | 16.7 (5.7-27.7) | 11.7 (8.0-15.4) | *p=0.560* |
| median time to MFP | 8.5 (5.8-11.2) | 9.9 (4.4-15.4) | 6.8 (4.2-9.4) | *p=0.837* |
| median time to BM | 7.9 (4.9-10.8) | 7.4 (2.1-18.2) | 7.8 (5.0-10.7) | *p=0.410* |
| median time to ecDM | 5.1 (2.9-7.3) | 6.0 (0.2-11.8) | 4.6 (1.8-7.3) | *p=0.656* |
| median time to LRP | 8.1 (6.1-10.0) | 7.5 (5.5-9.5) | 8.4 (6.1-10.7) | *p=0.383* |
| median time to death without progression | 15.4 (6.3-24.5) | 27.7 (13.7-41.7) | 11.1 (6.9-15.3) | *p=0.011* |
|  |  |  |  |  |
| **Post Progression survival (PPS)** | **months (95%CI)** | **months (95%CI)** | **months (95%CI)** |  |
| median PPS of all patients with progression | 12.6 (5.5-19.7) | 20.7 (17.7-23.8) | 8.9 (5.0-13.1) | *p=0.012* |
| median PPS after MFP | 18.6 (3.2-34.0) | 20.7 (0.0-41.6) | 10.3 (0.0-25.5) | *p=0.390* |
| median PPS after BM | 19.9 (10.5-29.5) | *not reached* | 12.4 (4.7-20.1) | *p=0.072* |
| median PPS after ecDM | 12.5 (0.00-26.0) | 22.7 (0.00-49.2) | 8.9 (2.3-15.6) | *p=0.155* |
| median PPS after LRP | 8.5 (7.5-9.4) | 7.8 (6.6-9.0) | 8.5 (8.1-8.8) | *p=0.588* |

Table 2 patient characteristics and results based on gender
