# Supplementary material for: Immune checkpoint inhibition alters patterns of failure in inoperable stage III non-small cell lung cancer patients treated with chemoradiotherapy
Source: J Cancer Res Clin Oncol. 2025 Nov 1;151(12):313. doi: 10.1007/s00432-025-06355-y (PMC12579610; doi:10.1007/s00432-025-06355-y)
Supplement: Supplementary file 2 — Supplementary Material 2 [file 432_2025_6355_MOESM2_ESM.docx]

**Immune checkpoint inhibition alters patterns of failure in inoperable stage III Non-Small Cell Lung Cancer patients treated with chemoradiotherapy**

Julian Taugner1,4 MD; Silja Stamer1; Kerstin Hofstetter MD2; Chukwuka Eze2,5 MD; Lukas Käsmann2,5 MD; Kerstin Clasen1 MD, Philipp Hartig1 MD; Werner Spengler3 MD; Thorben Groß3 MD; Farkhad Manapov,6 MD Claus Belka2,5 MD; Maximilian Niyazi1,2,4,5 MD.

*^1^Department of Radiation Oncology, University Hospital Tuebingen, Tuebingen Germany.*

*^2^Department of Radiation Oncology, University Hospital, LMU Munich, Munich
 Germany.*

*^3^Department of Medical Oncology and Pneumology (Internal Medicine VIII), University Hospital Tuebingen, Tuebingen Germany.*

*^4^National Center for Tumor Diseases (NCT), Partner Site Tuebingen, Tuebingen, Germany*

*^5^German Cancer Consortium (DKTK), Partner Site Munich, Munich Germany*

*^6^Radio-LOG MVZ Guenzburg, Guenzburg Germany.*

**Corresponding author:**Julian Taugner
Mail: [Julian.Taugner@med.uni-tuebingen.com](mailto:Julian.Taugner@med.uni-tuebingen.com)
ORCID: <https://orcid.org/0000-0003-1473-1202>
